# Family Physicians with Certificates of Added Competence in Palliative Care Contribute to Comprehensive Care in Their Communities: A Qualitative Descriptive Study

**DOI:** 10.1089/pmr.2022.0057

**Published:** 2023-02-16

**Authors:** Michelle Howard, Shireen Fikree, Ilana Allice, Alexandra Farag, Henry Yu-Hin Siu, Alison Baker, Jose Pereira, Shera Hosseini, Lawrence Grierson, Meredith Vanstone

**Affiliations:** ^1^ Department of Family Medicine, Innovation, and Theory (MERIT), McMaster University, Hamilton, Ontario, Canada.; ^2^ Division of Palliative Care, Innovation, and Theory (MERIT), McMaster University, Hamilton, Ontario, Canada.; ^3^ McMaster Education Research, Innovation, and Theory (MERIT), McMaster University, Hamilton, Ontario, Canada.

**Keywords:** Canada, certification, credentialing, family practice, palliative medicine, qualitative research

## Abstract

**Background:**

Since 2015, the College of Family Physicians of Canada has certified enhanced skills in palliative care (PC) with a certificate of added competence.

**Aim:**

This study aimed to describe the ways family physicians with enhanced skills in PC contribute within their communities, the factors that influence ways of practicing, and the perceived impacts.

**Design:**

Secondary analysis of data from a multiple case study on the role and impacts of family physicians with enhanced skills (i.e., PC physicians) was undertaken.

**Setting/Participants:**

Interviews were conducted in 2018 to 2019 with PC and generalist family physicians and residents associated with six family medicine practice cases across Canada. An unconstrained qualitative content analysis was performed.

**Results:**

Twenty-one participants (nine PC physicians, five generalist family physicians, two residents, and five physicians with enhanced skills in other domains) contributed data. PC physicians worked by enhancing their own family practice or as focused PC physicians. Roles included collaborating with other physicians through consultations, comanaging patients (shared care), or assuming care of the patient as the main provider (takeover). PC physicians increased capacity among their colleagues, with some patient care and education activities not being remunerated. Funding models and other structures were perceived as incentivizing the takeover model.

**Conclusion:**

Family physicians with enhanced skills in PC contribute to comprehensive care through the end of life. Remuneration should support system capacity and relationships that enable family physicians to provide primary PC especially outside the takeover model.

## Introduction

As life expectancy increases, more people are living longer with serious chronic conditions.^1^ End-of-life care is increasingly needed in health care systems. In Canada, approximately two-thirds of people die in hospital and fewer than one in six receive palliative home care.^2^ It is recognized that earlier and more integrated palliative care (PC) is needed,^2^ and this will require both specialist-level and primary-level PC services.^3^ The former is provided by clinicians with advanced training in PC who care for patients with complex needs, whereas the latter is provided by health care professionals across different settings and professions who, given basic training, can provide a PC approach to patients with less complex needs. Family physicians play a key role in providing primary PC supported by specialist-level PC physicians.^3,4^

Before 2016, palliative medicine was not a distinct specialty or subspecialty in Canada,^5^ although some physicians have large or exclusively PC practices. The proportion of Canadian physicians whose practice is extensively palliative care is estimated to be 1–3%.^2^ Since 1999, advanced PC training has been recognized by the College of Family Physicians of Canada (CFPC) and the Royal College of Physicians and Surgeons through a one-year added competency residency program after certification in family medicine or another specialty. In 2012, the CFPC introduced certificates of added competence (CAC) in designated clinical areas to recognize the acquisition of enhanced skills that support the comprehensiveness of the care family physicians provide within their communities.^6^

A CAC in PC (CAC-PC) was initiated in 2015 mainly through a one-year residency training program after family medicine certification, although the CFPC has also provided certification through a practice-eligibility route. By 2020, ∼600 CAC-PC designations had been awarded in Canada. In addition, there are family physicians who through other training or practice experience are considered to have enhanced skills in PC without the CAC-PC. In addition to enhancing the comprehensiveness of family medicine in their communities,^7^ physicians with CAC-PC work in various settings across the health care system and often provide specialist PC services in hospitals, PC units, and long-term care facilities.

In a previous multiple case study, we investigated the impact of enhanced skills family physicians, including those with CACs (care of the elderly, PC, anesthesia, and sports medicine) in family physician groups in Canada. The report provided an overarching picture of enhanced skills and CAC-holder physicians but did not highlight unique features of PC.^7^ In this study, we report a secondary analysis of qualitative data to describe the ways in which family physicians with a CAC-PC or other enhanced skills in PC work in their communities, to identify factors that influence how these physicians work, and the perceived impacts of this work.

## Methods

### Study design

The methods of the primary study have been reported previously.^7^ In brief, between September 2018 and June 2019, we conducted six instrumental case studies.^8,9^ In instrumental case studies, the cases are studied to provide insight into a specific issue, and in this study the goal was to develop a framework of relevant factors and their relationships to CAC-mediated comprehensive care delivery.

### Case selection and participants

We defined cases as a collective of family physicians working with a defined group of patients in an interconnected community. Cases from any Canadian province or territory were eligible, and we sampled cases to achieve maximum variation across geography, population density, language, patient population, and practice model. To identify cases, we reviewed the distribution of CAC physicians from CFPC data, reviewed family practice websites, and connected by e-mail with family physicians knowledgeable about practices in particular regions then invited regional representatives to discuss the family medicine context in their area and nominate a practice group that exhibited desired features.

Eligible participants were any professional whose work was related to the case, including CAC holders, other enhanced skill family physicians, generalist family physicians, resident trainees, physicians in other specialty areas, and administrative staff. The designation of enhanced skills could be self-identified or credentialed. These were CFPC-accredited physicians who had, through training or the accumulation of experience, acquired extra clinical expertise and were providing services in a defined domain of care.

We combined purposeful, criterion, and snowball sampling techniques and ceased recruitment when data sufficiency was determined, related to data completeness within each case.^9^

### Data collection

All interviews were conducted at the participant's workplace or by telephone by a female masters-trained qualitative researcher employed as a research assistant. The interview guide was developed based on key issues identified in the literature as described previously,^7^ by our CFPC partners, and the research team, and pilot tested. Participants were aware that the interviewer was conducting research funded by the CFPC concerning the CAC program. Interviews were designed to last one hour and were audio-recorded and transcribed verbatim. Fieldnotes were taken. No participants withdrew from the study. Transcripts and findings were not returned to participants for comment.

### Data analysis

We extracted data that were related to PC from the interviews. Qualitative description was chosen because our aim was to attain a condensed and broad description of the phenomenon of how family physicians with enhanced skills in PC contribute to their communities. We used an unconstrained deductive analytic approach because our analysis was grounded in the models that arose from the main study conceptualizing the ways physicians with CAC work generally. We also used an inductive content analysis approach to elaborate the description of PC. The results of our previous study provided the initial framework for coding and analysis.^7^

An unconstrained deductive approach allows us to refine, expand, or adapt the framework to fit the data specific to the provision of PC. Three analysts were involved (S.F., M.H., and S.H.). First, two analysts (S.F. and M.H.) worked independently using a deductive approach to code data on the ways enhanced skills physicians with and without CAC-PC designation (hereafter referred to as PC physicians) worked according to the previously identified models. Concurrently, they created additional codes for descriptions that differed from or augmented the previous models. After the two analysts reached consensus on the deductive coding, M.H. and S.H. inductively coded factors influencing the way PC physicians worked in their communities and their impacts.

Data were managed through N-Vivo12 (QSR International Pty Ltd. (2020) and NVivo [March 2020]). This study was approved by the Hamilton Integrated Research Ethics Board (HiREB no. 5151, September 2018). All participants provided written and verbal informed consent.

## Results

Six cases and 21 participants contributed data. Participant characteristics are summarized in Table 1. Of 21 participants, six held CAC-PC credentials (denoted as CAC-PC in quotes) and three identified themselves as enhanced skills physicians in PC but without a CAC designation (denoted as non-CAC-PC in quotes). Table 2 provides participant quotes representative of the themes hereunder.

**Table 1.** Characteristics of Participants Who Described the Role of Enhanced Skills and Certificate of Added Competence Credentialed Palliative Care Physicians in Their Communities

Case	Participant	Role	Sex	Geography
1	1	Non-CAC-multiple domains of care	Female	Prairies/rural
	4	Non-CAC COE	Male	
	6	Non-CAC-PC	Male	
	7	Addiction medicine	Male	
	8	CAC-PC	Male	
2	1	Non-CAC generalist	Female	Atlantic/urban
	3	Non-CAC generalist	Female	
	4	CAC-PC	Female	
	5	Non-CAC resident	Female	
	7	Non-CAC generalist	Female	
3	1	CAC-PC	Male	Northern territories/remote
4	5	Non-CAC generalist	Female	Pacific/rural
	7	Non-CAC resident	Male	
	8	Non-CAC PC	Female	
5	1	Non-CAC generalist	Male	Central/urban
	2	CAC-PC	Male	
	3	COE	Male	
	4	CAC-PC	Male	
	5	Non-CAC PC	Female	
6	1	COE	Male	Central/suburban
	4	CAC-PC	Female	

CAC, certificates of added competence; COE, care of the elderly; PC, palliative care.

**Table 2.** Thematic Organization of Representative Participant Quotes

Theme	Quote
Models of physician practice	
Mixed	we actually have talked about that a fair number of times in our office where we talked about the idea of having a smaller group of our family docs take over the care of anyone who is part of our family health team. [Case 5 #3, CAC care of the elderly]
PC focused	…where the communities don't have family docs, we're often the primary resource for patients with palliative diagnoses who we've already seen to manage them from afar. Because those people regularly get their primary health care through visiting clinics, docs have contracts to go out for one day a month or one week a month type deal… [Case 3 #1, CAC-PC] [Referring to program for structurally vulnerable people]…Obviously, we're a palliative care team, but people can have depression, people can have poverty, people can have a fungal toe infection. So, we are practicing at a general level. While most people have palliative care needs, they also have primary care needs. [Case 5 # 2, CAC-PC]
Models of patient care	
Collaborative-consultation	At least, you have that back-up, where you get the help. Because, some specific symptoms are just hard to deal with. Like, seizures. Some symptoms that you don't see often, but the patients are suffering, the families are panicked, and it's really important to get those symptoms managed as soon as possible. To have somebody that you can just call and get the advice right away, rather than having to read up and look up and try to figure it out yourself and not really feel comfortable. [Case 2 #3, non-CAC generalist]
Collaborative-shared care	That's very rare, where we actually take over as MRP (most responsible physician). That happens sometimes in hospice, but we try to only act in a consulting role. That being said, sometimes it's really hard, because there will be complicated things and then the GP, and I get it, because I was doing family practice for a long time. The physicians here are doing hospital care, rounding in the morning, trying to make it to their office. It's a lot easier to say, let's let the palliative care team sort this out. But, a lot of the stuff, then, we end up kind of, how involved do we get. Stuff gets really complicated and then you end up taking on more of the MRP role. [Case 4 #8, non-CAC PC] I will be usually asked to see a patient that a most responsible physician and/or a resident are struggling with either symptom management or important conversations that need to be had [Case 6, #4 CAC-PC, regarding role in participant's own multi-physician family practice] In the case of nephrology, we actually have an embedded palliative care clinic within their kidney care clinic. So, a palliative care physician is sitting beside a kidney care nurse is sitting beside a nephrologist… With the oncology group, we've been able to build in-roads for particular pathways, certain malignancies that have great evidence for palliative care delivery and improvement of outcomes. For example, lung cancer or pancreatic cancer are really direct pathways toward palliative care delivery. In the case of COPD, we've built criteria with our respirologist team, to help them identify people who may benefit from early palliative care needs. In one case, we actually are physically in their clinic, in others we've built formal criteria for particular disease groups. [Case 5 # 2, CAC-PC]
Takeover	But when it comes to really reaching that end of life, personally, I do prefer when I am able to completely take over that care just because….it is easier to a) build that rapport and b) make sure that everyone is on the same page with what the plan is and what's happening and to manage symptoms… [Case 5 #5, non-CAC-PC]
Capacity building for palliative care	..he [PC physician] talked to us about things like standard orders. He instituted several things that he learnt in … that he translated to here. He made a little card that had some of the basics about opioids and how you convert Fentanyl to Morphine or the other way around. It had ideas on nausea and some of the basic symptoms that patients go through in palliative care. Now, you can rattle them off. The p.r.n. orders, there's probably a standard order form now for those. [Case 1, #7 Addiction Medicine] And, I think the CAC program provides us, as continuing trainees, a little bit of expertise in that specific field and taking on more leadership roles and academic roles, in terms of furthering the field that way. [Case 5 #4, CAC-PC]
Factors influencing practice	
Salaried-type remuneration	The palliative care fee structure supports me spending as much time as I need with someone so that's why I'll go drive half an hour out of town to spend an hour with someone and then drive back and not worry about it, or I'll text them, or phone call them so that type of thing. [Case 3 #1, CAC-PC] …we actually have an alternative payment plan contract with the health authority, so that allows us to do education, it allows us to do administration, it allows us to do indirect patient care as well as direct patient care. [Case 4 #8, Non-CAC-PC]
Fee-for-service remuneration	…you cannot do shared care and have both doctors paid at the same time in the model that we're in. …. So, if a family doctor wants to do shared care, obviously they're going to bill for it, that's kind of the point and the incentive, so we kind of work for free in those cases. [Case 6 #2 CAC-PC]
Collaboration processes	… there needs to be improved understanding of what early palliative care needs are. I'm not saying we get zero referrals from family doctors, but a lot of our referrals, the majority come from specialists and from people who have ended up in hospital because they're already too sick….my experience has been that primary care practitioners are not trained, like they're trained about when to refer to a cardiologist or a respirologist. There seems to be comfort with that, but when it comes to referrals to early palliative care interventions, it doesn't seem that this is well understood. So, generally, we're not getting referrals from family physicians early. [Case 6 #2, CAC-PC]

COPD, chronic obstructive pulmonary disease; GP, general practitioner; MRP, most responsible physician.

### Models of physician practice

The main distinguishing feature of how PC physicians practiced was their focus of practice. Some participants described a “mixed” approach where the PC physician maintained a generalist family practice and provided PC alongside primary care to their own patients or to other patients in their communities. Participants described the benefit of allowing patients who required PC to remain in their own family practice, avoiding referral elsewhere. It was also noted that a PC physician working this way may have had more patients needing PC, such as patients with cancer, than would be typical in a family practice.

Alternatively, PC physician practice was characterized as “palliative care focused” in which they did not generally provide primary care and instead worked in PC focused teams or services across various settings and as part of PC teams through specific arrangements in their communities. Clinically their practice was predominantly or in some cases entirely PC; however, there were circumstances where PC physicians employed their general family medicine knowledge. One PC physician working in a remote region described being called by the nurse to consult on general primary care issues, and in an urban outreach PC team, the PC physician provided general primary care for individuals who are structurally vulnerable (e.g., without homes).

### Models of patient care

Within the mixed and PC-focused practice styles, participants described different ways PC physicians provided patient care. These arrangements were defined by the respective roles of the PC physician and most responsible physician (MRP) and exhibited various degrees of collaboration and responsibility in the context of a given patient. Two broad models of interaction were apparent: a “collaborative” model and a “takeover” model.

The collaborative model was defined by PC physicians providing support to family physicians without assuming overall care of the patient. The collaborative model included both “consultation” and “shared care.” Participants described consultations on specific clinical issues such as challenging symptoms they were trying to manage, with or without a consultation with the patient.

The consultation model sometimes evolved to a “shared care” model, where the family physician comanaged the patient with the PC physician. This was sometimes noted to occur when the complexity of the patient increased.

“Whether that's working in a consultative way with symptom management or if it's have the family physician, give them a little bit of direction around those things and lay off for a little bit and then if there become complex needs, be reconsulted and join the patients care.” [Case 5 no. 4 CAC-PC].

PC physicians with a generalist family practice (mixed practice) did not necessarily only provide PC to their own patients. They also engaged in “collaborative care” with respect to other family physicians.

“…we talked about the idea of having a smaller group of our family docs take over the [palliative] care of anyone who is part of our family health team” [Case 5, no. 3, CAC-care of elderly]

Although the study was centered on six family practice cases, the role of PC physicians in other specialties was noted. The collaborative model of PC physicians extended beyond family practice to outpatient specialist clinics such as cancer or nephrology clinics.

“In the case of nephrology, we actually have an embedded palliative care clinic within their kidney care clinic. So, a palliative care physician is sitting beside a kidney care nurse is sitting beside a nephrologist…” [Case 5 no. 2 CAC-PC]

In the takeover model, the patient is transferred to the PC physician, who becomes the MRP until the patient's time of death. Participants noted the positive impacts of the takeover model, including better coordination with a single most responsible provider, the ability to build rapport with the patient, and the expertise to manage complex issues.

Typically, it tends to be a complete transfer of care from the family physician to us, just because as I mentioned, we go out to see our palliative patients quite regularly. It can be very confusing for that completeness of care if that patient is coming to see us, but then also is still travelling to go see a family physician, as well, because then things get lost in translation. One person starts them on this medication and another starts on this medication. It does not lead to very good care in the end. Typically, we do end up taking over [Case 5 no. 5, non-CAC-PC].

### Capacity building for PC

PC physicians increased capacity for PC among generalist family physician colleagues and other specialists in two main ways. Participants described learning through consultations with PC physicians regarding specific patients, which could be applied to other patients. They also described how PC physicians provided general education and tools to their generalist colleagues. CAC-PC physicians noted their academic and leadership roles, and it was noted that secondary or tertiary academic centers would expect the CAC credential.

…one of the things that the CAC, as I'm now exploring different job opportunities and different academic positions, I think it provides you with almost a vetting, that you have the experience with the leadership, with the field. [Case 5, no. 4, CAC-PC]

### Factors influencing practice

Remuneration models, whether fee-for service (in which physicians billed health ministries for clinical work) or salaried-type arrangements, influenced the models of practice of the PC physicians. The lack of need (and ensuing lack of billing opportunities or salary-type funding) to support a full-time PC physician was a factor contributing to family physicians maintaining their own practice and leveraging their enhanced skills to provide PC. Sometimes special blended models or sessional funding arrangements were negotiated regionally.

Salaried remuneration was noted to facilitate PC physicians' ability to support patient needs by allowing time for longer visits and home visits. Salaried remuneration also allowed PC physicians to engage in beneficial nonclinical education and administration activities.

Fee-for-service payment was considered challenging because both the family physician and PC physician cannot simultaneously bill PC fees for a patient concurrently, thereby encouraging the takeover model and discouraging collaboration models. Family physicians were perceived to be sometimes reluctant to hand over patients until late in the patient's trajectory because some billing codes penalized the family physician for having the patient seen by another family physician (i.e., when the PC physician had a family medicine designation). PC physicians were noted to sometimes provide consultations to family physicians without remuneration.

The absence of processes such as referral pathways for family physicians also influenced involvement of PC physicians. This situation was contrasted to the use of referral pathways by specialist clinics and hospitals who refer directly to PC. Sometimes it was perceived that family physicians were reluctant to seek consultation of a PC physician because they did not recognize a need.

Especially working in a smaller community, there are many doctors here who think that they can do everything. And, they do a pretty good job of it most of the time, but sometimes there are physicians that do not recognize what their limitations are and you cannot really be an expert in everything all the time, even though some people think that they are. So, there are people who definitely are not getting good PC, they do not die well at all, because their physicians will never consult us, because they never want to ask for help. [Case 4 no. 8, non-CAC-PC]

## Discussion

In this qualitative study of six family practices and 21 individuals, the roles of the PC physician were described as acting as a consultant, sharing care with the MRP, or taking over care of the patient until the end of life. Similar to other CAC domains,^7^ this study found that some physicians choose not to practice comprehensive family medicine and instead focus their practice on their area of enhanced skill. Reasons for practicing in specific ways were described in the context of local needs, systems and processes, and remuneration mechanisms. PC physicians also integrated into specialist disease acute care hospitals and services, outpatient hospital clinics, and family health clinics, demonstrating their broad range of practice throughout the system. Impacts of PC physicians included meeting PC needs across communities and building capacity among generalists.

The results of this study align with previously described models of PC physician practice on a spectrum from a consultation model supporting generalists, to a takeover model where the PC physician assumes the role of MRP.^10–13^ We found that handover of the patient to a PC physician was related to comfort and skill levels among generalists, and the complexity of patients. Late referrals to PC physicians were noted. Fee-for-service remuneration models for PC physicians were not conducive to supporting collaborative models, whereas the lack of supporting structures and resources were perceived not to support primary-level PC by generalists. PC physicians were able to support a collaborative model through informal and formal consultations with generalist family physicians and by capacity building through education. In some instances, these activities were undertaken by individuals in fee-for-service models that reimburse only clinical work.

This study has implications for policy and future research. Through collaborative and shared care models, PC physicians promoted primary-level PC capacity building across the Canadian health care system. In some cases, PC physicians took over care as most responsible clinicians especially when patients' needs were complex. This is an important contribution to the health care system. Of concern, however, is the possibility that CAC physicians take over the provision of primary PC from primary care colleagues and colleagues in other specialty areas, thereby losing the opportunity to spread core PC competencies and thereby increase access to PC.^14^ The collaborative care model has other advantages, including ensuring continuity in the patient's relationship with their existing family physician.^15^ Continuity with the family physician is associated with less use of acute care services at the end of life.^16–18^

The collaborative care model also allows generalist family physicians to enhance their knowledge and skills,^11^ and could help to address the limited exposure to generalist PC in family medicine residency training.^19,20^ There are variations across Canada in how PC physicians provide PC in community settings; in some provinces the consultation model appears to predominate, whereas in others the takeover model does.^21–23^ Examples of integrated approaches to support generalist family physicians in providing PC have been successfully developed in Canada,^11,24,25^ but challenges remain in consistently implementing these approaches. Research is needed to understand how policies relating to funding and health human resources support or hinder integration.^26^ Ensuring there is exposure to collaborative PC models and approaches during training may further enhance the spread of core PC competencies.

### Strengths and limitations

A strength of this study is the inclusion of perspectives from different physician types practicing in diverse settings across Canada. The study was designed to investigate several practice domains in the context of comprehensive family medicine and did not explicitly address PC models in all settings where PC physicians practice, or other roles of CAC holders in academic centers in teaching, scholarship, and leadership. Data were insufficient to examine whether models differed between physicians with the CAC-PC versus those who attained enhanced PC skills through other mechanisms.

## Conclusion

Physicians with enhanced skills designations in PC worked in their communities to facilitate comprehensive care of people throughout the PC trajectory. They practice in collaborative models, takeover models, and by building capacity among other providers. To facilitate the goals of the Family Medicine CAC-PC in fostering comprehensive care in communities, remuneration models should support system capacity and relationships that enable family physicians to provide primary PC, especially outside the PC physician takeover model.

## Abbreviations Used

CAC: certificates of added competence
CFPC: College of Family Physicians of Canada
COE: care of the elderly
COPD: chronic obstructive pulmonary disease
GP: general practitioner
MRP: most responsible physician
PC: palliative care
